# GETTING OLDER ADULTS INVOLVED IN CLIMATE CHANGE INITIATIVES: REFRAMING AGING AS A POTENTIAL APPROACH

**DOI:** 10.1093/geroni/igad104.0498

**Published:** 2023-12-21

**Authors:** Manfred Diehl

**Affiliations:** Colorado State University, Fort Collins, Colorado, United States

## Abstract

In the context of climate change, older adults are often solely portrayed in terms of vulnerability and victimization. Although older adults’ vulnerability to the growing effects of climate change and natural disasters is an important topic, this presentation focuses on how to get older adults involved in climate change initiatives and as advocates for the adoption of climate-friendly behavior. The presentation argues that getting the growing population of older adults actively involved in issues of climate change requires moving away from a deficit- and loss-focused view of later adulthood. Instead, a new narrative about aging needs to be adopted, recognizing the many contributions older adults can make to addressing the global climate crisis. These contributions arise out of concerns for the health and well-being of the next generations and draw on older adults’ legacy thinking, lifelong experiences, professional expertise, and ability to work in multigenerational contexts. Such a new narrative recognizes and supports older adults as a “natural resource” and important “human capital.” The presentation will conclude with a discussion of the challenges to get older adults involved in climate change initiatives and with a description of several exemplary programs that have already generated foundational knowledge in this domain.

